# Nitrosylated Hemoglobin Levels in Human Venous Erythrocytes Correlate with Vascular Endothelial Function Measured by Digital Reactive Hyperemia

**DOI:** 10.1371/journal.pone.0076457

**Published:** 2013-10-10

**Authors:** Irina I. Lobysheva, Pauline Biller, Bernard Gallez, Christophe Beauloye, Jean-Luc Balligand

**Affiliations:** 1 Institut de Recherche Experimentale et Clinique (IREC), Pole of Pharmacology and Therapeutics (FATH), Université Catholique de Louvain (UCL), Brussels, Belgium; 2 Louvain Drug Research Institute, Biomedical Magnetic Resonance Unit, UCL, Brussels, Belgium; 3 Pole of Cardiovascular Research (CARD), and Departments of Internal Medicine and Cardiovascular Diseases, Cliniques Universitaires Saint-Luc, Université Catholique de Louvain, Brussels, Belgium; Bristol Heart Institute, University of Bristol, United Kingdom

## Abstract

Impaired nitric oxide (NO)–dependent endothelial function is associated with the development of cardiovascular diseases. We hypothesized that erythrocyte levels of nitrosylated hemoglobin (HbNO-heme) may reflect vascular endothelial function *in vivo*. We developed a modified subtraction method using Electron Paramagnetic Resonance (EPR) spectroscopy to identify the 5-coordinate α-HbNO (HbNO) concentration in human erythrocytes and examined its correlation with endothelial function assessed by peripheral arterial tonometry (PAT). Changes in digital pulse amplitude were measured by PAT during reactive hyperemia following brachial arterial occlusion in a group of healthy volunteers (50 subjects). Erythrocyte HbNO levels were measured at baseline and at the peak of hyperemia. We digitally subtracted an individual model EPR signal of erythrocyte free radicals from the whole EPR spectrum to unmask and quantitate the HbNO EPR signals.

**Results:**

Mean erythrocyte HbNO concentration at baseline was 219+/−12 nmol/L (n = 50). HbNO levels and reactive hyperemia (RH) indexes were higher in female (free of contraceptive pills) than male subjects. We observed a dynamic increase of HbNO levels in erythrocytes isolated at 1–2 min of post-occlusion hyperemia (120+/−8% of basal levels); post-occlusion HbNO levels were correlated with basal levels. Both basal and post-occlusion HbNO levels were significantly correlated with reactive hyperemia (RH) indexes (r = 0.58; P<0.0001 for basal HbNO).

**Conclusion:**

The study demonstrates quantitative measurements of 5-coordinate α-HbNO in human venous erythrocytes, its dynamic physiologic regulation and correlation with endothelial function measured by tonometry during hyperemia. This opens the way to further understanding of *in vivo* determinants of NO bioavailability in human circulation.

## Introduction

Impairment of nitric oxide (NO) bioavailability is a hallmark of endothelial dysfunction in cardiovascular and metabolic diseases associated with hypertension, atherosclerosis, diabetes, hypercholesterolemia and aging [1], [2]. Vascular NO is produced by the endothelial NO synthase (eNOS), a tightly regulated enzyme, and plays a key role in vascular homeostasis. However, the bioavailability of NO is influenced by the concurrent production of reactive oxygen species and the redox status in the vasculature [3], [4]. A reliable assay of the bioavailability of NO in human circulation *in vivo* would be highly desirable for monitoring the progression and the prevention of cardiovascular diseases as well as treatment tailoring with newly-developed drugs targeting endothelial function.

Analytical methods have been developed to measure the level and dynamics of NO bioavailability using invasive and non-invasive approaches. Measurements of circulating nitrite/nitrate, of nitrosylated proteins, of cGMP and phospho VASP content in tissue biopsies, or stable isotopic methods have been used in human studies with some limitations of specificity/sensitivity and all are affected by confounding factors limiting the interpretation of the results [5], [6]. On the other hand, non-invasive methods have been developed to evaluate NO-dependent endothelial function, including measurements of flow-mediated dilation (FMD) by ultrasonic scanning and of peripheral vasodilator response using fingertip pulse amplitude tonometry (PAT) during reactive hyperemia [7]. Clinical studies of large population cohorts have demonstrated significant correlations of these functional parameters with multiple traditional cardiovascular risk factors typically associated with endothelial dysfunction (ED) [8], [9], [10]. However, none of these provided a quantitative measurement of circulating levels of NO, a key mediator of this endothelium-mediated vasodilation and vascular homeostasis.

One of the most powerful techniques to analyze NO bio-production is Electron Paramagnetic Resonance (EPR) spectroscopy, a method for the quantitative detection of paramagnetic molecules. Nitric oxide itself is paramagnetic, but its detection in biological tissues and liquids remains a challenge because of low concentration, short half-life and characteristics of the EPR signal (reviewed in [11], [12]). In circulating blood, the reaction of NO with hemoglobin is predominant with rate constants in the range of 2×10^7^ to 1×10^8^ M^−1.^s^−1^ depending on the hemoglobin environment and the oxygenation state. Monitoring of paramagnetic heme-nitrosyl adducts of hemoglobin by EPR spectroscopy is attractive due to potential quantitative information about the fate of NO in human blood. Indeed, at least three paramagnetic forms of nitrosylated Hb were observed in human and rodent blood at different Hb conformations and NO-hosting subunits: 5-coordinate α-HbNO (T-form, deoxy-like); 6-coordinate-α-HbNO (R-form, oxy-like); 6-coordinate-β-HbNO (R-form, oxy-like). These adducts exhibit EPR signals with different characteristics due to corresponding electronic configurations. The first two forms were predominantly observed in freshly frozen blood of rodents due to low stability of nitrosylated β-heme adducts. Remarkably, a typical EPR spectrum of 5-coordinate nitrosyl-heme (α-Hb, T-form, predominantly observed in venous blood, referred to as HbNO in the following text) displays the well-resolved triplet hyperfine (hf) structure due to net donation of electron density from Fe(II) to NO after cleavage of the bond between the heme iron and the proximal His residue of the R-form (reviewed in [11], [13]). However, accurate interpretation of EPR signals in human blood has remained a challenge due to overlapping EPR signals originating from other paramagnetic species, combined with low basal concentrations of HbNO [14]. Very few articles so far reported on EPR analysis of HbNO in human blood, e.g. after administration of hydroxyurea [15], [16]. Accurate separation of different forms of nitrosylated Hb, circulating in arterial and venous human blood after NO gas inhalation, was demonstrated using regression-based spectral analysis, but the low basal level of nitrosylated Hb detected by EPR in whole human blood hampered the use of the technique in subsequent studies [17].

We developed a new approach specifically designed to increase the sensitivity of detection of circulating HbNO *in vivo*. In the present study, we describe a modified EPR subtraction method to identify the T-form of HbNO in fast-spun erythrocytes from human venous blood. Using this technique, we further examined whether the measured levels of HbNO correlate with endothelial function assessed from the peripheral vasodilator response to reactive hyperemia in a group of healthy volunteers.

## Materials and Methods

### Ethics Statement

The study protocol was approved by the Biomedical Ethical Committee of the Faculty of Medicine and St. Luc university hospital of the Université Catholique de Louvain. (approval number: 2011/26AVR/161-belgian registry: B403201111288). All subjects signed an informed consent.

### Study Subjects

Fifty volunteers were individually informed about the modalities of the study, including measurement of endothelial function using finger plethysmography with a peripheral arterial tonometer (Endo-PAT, Itamar, IL), venous blood sampling for EPR spectroscopy and routine laboratory analysis for the assessment of cardiovascular risk factors (total, HDL, LDL cholesterol, and triglyceride levels, concentrations of homocysteine and C-reactive protein, CRP). All subjects were reportedly healthy and fasted since the night before the experiment. All tests were performed in the morning in a quiet environment with stable temperature by specifically trained personnel. The following parameters were recorded for each volunteer: sex; presence of hypertension; dyslipidemia; diabetes mellitus; smoking history; regular exercise; use of vitamin supplements or specific medications, including contraception pills for females.

### Assay of Digital Hyperemia Response by Fingertip Peripheral Arterial Tonometry

**Figure 1:**
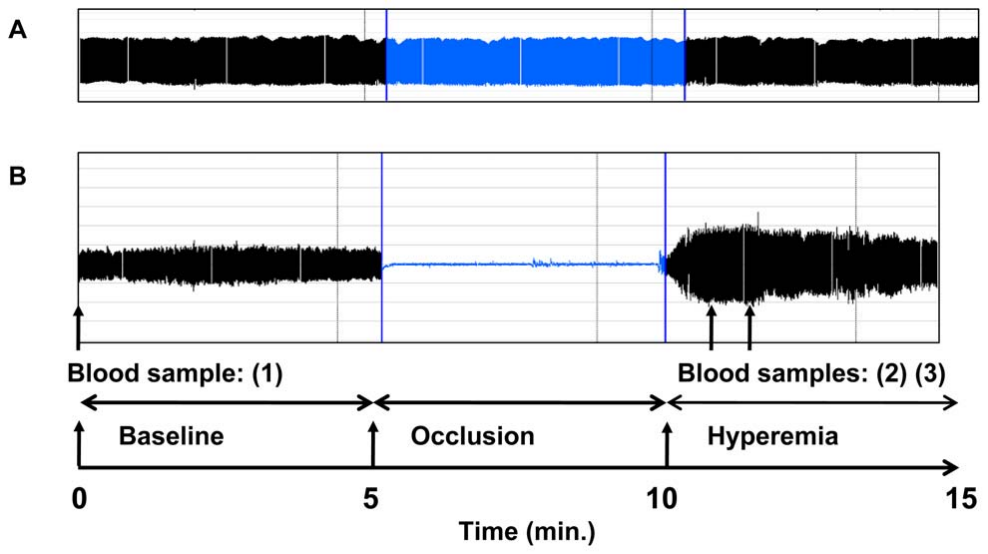
Study design. Combination of finger plethysmography (peripheral arterial tonometry, EndoPAT) with venous blood sampling from the same arm for EPR measurements of HbNO level. Changes of pulse amplitude, recorded by EndoPAT probes at baseline, during arterial occlusion (with a brachial cuff), and during reactive hyperemia (after cuff deflation) at the tip of the index finger of the ischemic arm (B) and contralateral finger of control arm (not undergoing reactive hyperemia) (A). Blood sampling for HbNO EPR assay was done at baseline (sample 1) before EndoPAT signal recording and at 1 (sample 2) and 2 (sample 3) minutes post-deflation of the cuff (post-occlusive hyperemia).

Digital hyperemia response was measured at finger (index) tips using an EndoPat2000 device (Itamar Medical). Briefly, pulse wave amplitude (PWA) changes were assessed as beat-to-beat plethysmographic signals in the index finger by high-sensitive pneumatic probes (EndoPAT™, Itamar). The signals were measured from each fingertip at basal state during 5 minutes. Then brachial blood flow was interrupted for 5 minutes by inflation of a sphyngomanometer cuff placed on the proximal forearm of the non-dominant hand, and PWA signals were recorded during occlusion and after restoration of blood flow for 10 minutes. Typical tracings are represented in Figure 1. Data were digitized and computed automatically by EndoPat2000 software; the following indexes characterizing endothelial function were calculated: the Framingham reactive hyperemia index (FRHI) defined as the natural logarithm of the ratio of mean post-deflation signal (in the 90 to 120-second post-deflation interval) to baseline signal in hyperemic finger normalized by the same ratio in the contra-lateral finger; or the reactive hyperemia index (RHI) defined as the ratio of mean post-deflation signal (in the 90 to 120-second post-deflation interval) to baseline signal in hyperemic finger normalized by the same ratio in the contra-lateral finger and multiplied by a baseline correction factor (K = 0.52397×log(mean baseline amplitude)−0.2), as calculated by the EndoPAT software. FRHI was used in our study for correlation analysis as was previously performed in the Framingham study [8].

### Sample Preparation for Detection of Heme-nitrosylated Hemoglobin in Human Erythrocytes by EPR Spectroscopy and Experimental Design

Human blood was collected from the median cubital vein using a catheter (Eclipse, 21 gauge) into BD vacutainer tubes containing EDTA (1.8 mg/mL); sampling was done at baseline (i.e. before artery occlusion), then one and two minutes post-deflation (i.e. after restoration of blood flow – schematic representation of the protocol is shown in Figure 1), except in 4 subjects in which post-occlusion blood could not be obtained. Blood was immediately centrifuged at 800×*g* for 10 minutes (at temperature ∼10°C), then plasma was removed, and one aliquot of pelleted red blood cells (RBCs) was transferred into tubes calibrated for EPR measurements, immediately frozen, and stored in liquid nitrogen. Additional samples were further processed as described below. Low-temperature EPR spectra from the frozen samples were recorded, using an EPR quartz finger Dewar filled with liquid nitrogen at 77 K, on a Bruker EMX100 X-band spectrometer with the following setting: microwave frequency ^∼^ 9.35 GHz; modulation frequency, 100 kHz; microwave power, 20 mW; modulation amplitude, 7 mT; 10 scans.

### Preparation of Samples for Calibration Curve of HbNO Quantification Using NO-donor System In Vitro

For the characterisation of the EPR spectra, a calibration curve was generated using HbNO complexes synthesized in isolated RBCs after incubation with a mixture of sodium nitrite at different concentrations and sodium dithionite (Na_2_S_2_O_4,_ 20 mM) in anaerobic condition (1% of O_2_; RUSKINN workstation INVIVO_2_400; 37°C). In some experiments, Dea-NONOate (2-(N,N-Diethylamino)-diazenolate 2-oxide, purchased from Enzo Life Sciences), a NO-donor, was used to synthesize HbNO. In this case, small aliquots of Dea-NONOate solution (in 0.01N NaOH) were added to freshly isolated RBCs in anaerobic condition to a final concentration of 50 µmol/L. The samples were transferred into the calibrated tubes, and immediately frozen. The EPR spectra were recorded as described above. Strict linearity was observed for the proportional increase of either the intensity of HbNO EPR signal obtained by signal double integration, or the amplitude of first hf component with increasing concentrations of added nitrite (Figures **S**1A and S1B). The double-integrated intensities of the EPR signals were normalized by that of known EPR standards (Cu–EDTA complex, 50 and 100 µM frozen in 30% glycerol-water solution).

### Preparation of Samples for Digital Generation of Individual Model EPR Spectra and the EPR Signal Subtraction Procedure

**Figure 2:**
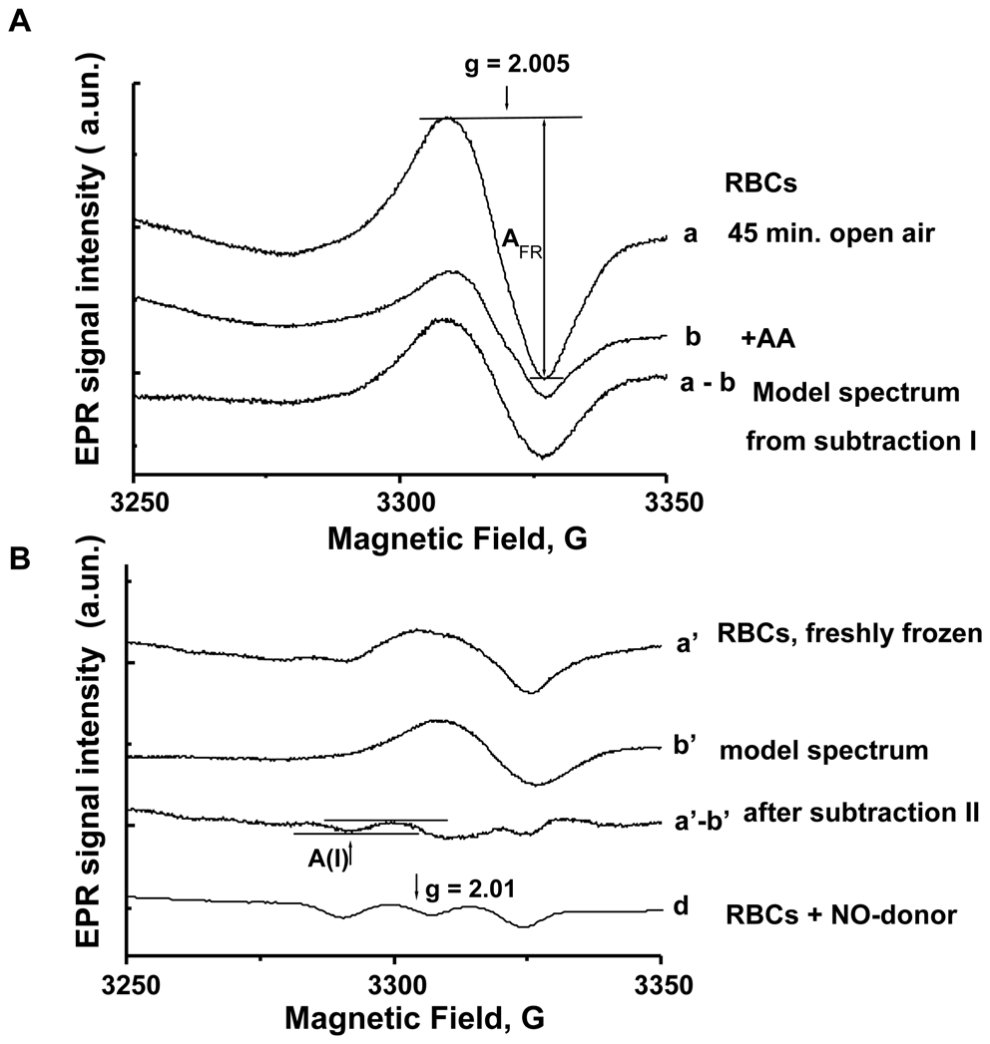
Measurements of HbNO in human RBCs by a modified EPR subtraction procedure. (A) Typical EPR spectra of free radicals in RBCs left for 45 min. in open air at room temperature: the individual model spectrum of free radicals in RBCs (*lowest spectrum, a–b*) was obtained after subtraction (subtraction I) of the EPR spectrum of RBCs treated with ascorbic acid (AA, 10 mmol/L, for the last 15 minutes) (b), from the EPR spectrum of RBC treated similarly with solvent (a). (B) Measurements of HbNO level in RBCs isolated from venous blood by EPR spectroscopy using subtraction (subtraction II) of individual free radical signal. The high field components of the EPR spectrum of RBCs frozen immediately after sampling (upper spectrum, a′) are shown at diapason 3250–3350 Gauss. The final EPR spectrum (a′–b′) shows the hf components of g_z_ HbNO signal after subtraction of the model EPR spectrum of free radicals (b′), divided by the correspondent proportional scale factor (gain = 6.23×10^4^); a typical EPR spectrum of RBCs, frozen after incubation with NO-donor (Dea-NONOate, 50 µmol/L) under low O_2_ level (gain = 0.62×10^4^) is shown for comparison (d). The peak-to-peak amplitude of the 1st hf component (A(I)) of the triplet hf structure of the EPR spectrum, attributed to 5-coordinate α-HbNO, was used for quantitation.

The EPR signal of the 5-coordinate alpha nitrosyl-heme is characterized by a three-line hf structure with coupling of 16.8 G in diapason near g-factor 2.01 (Figure S1A). It is overlapping with an EPR signal with g ∼ 2.005 originating from protein-centred free radicals [18]. To unmask the hf components, we developed a method using digital subtraction of an individual model spectrum of free radical (FR) signal, generated for each subject from two additional blood samples prepared as follows; i) first, two RBC sub-samples (collected as described above) were exposed to open air at room temperature for 30 minutes to allow disintegration of the intrinsic HbNO signal; ii) one sample was treated with ascorbic acid (AA, 10 mmol/L- or vitamin C, used as anti-oxidant) for a subsequent 15 minutes; iii) and the second sample was treated with vehicle (saline solution) identically. Then, both samples were frozen in liquid nitrogen and processed for EPR analysis. The difference spectrum resulting from digital subtraction between the two EPR spectra from these sub-samples (with/without anti-oxidant treatment; subtraction I) shows the corresponding signal of the protein-centred FR with individually fluctuating peak-to-peak width ∼ 16–18 G and was used as an individual model of FR signal (model spectrum; Figure 2A). This individually-determined model FR signal was then subtracted from the basal EPR signal of the RBC sample frozen immediately after centrifugation using a proportional scale factor depending on the amplitude of the FR signal (subtraction II; Figure 2B) to reveal the hyperfine structure of the HbNO spectrum. In some cases, N-acetyl-cysteine (10 mM, 15 minutes) or α-tocopherol (5 mM, 15 minutes) were used instead of ascorbic acid, with similar results (data not shown). Notably, ascorbic acid at the applied concentration of 10 mM reduced the protein free radicals signal of isolated RBCs in 53% of subjects. In the remaining subjects, ascorbic acid increased the free radical signal (a paradoxical “pro-oxidant” effect). In the latter, reverse subtraction was used to obtain the differential component corresponding to free radicals (Figure S2).

### Statistical Analysis

The analysis of the EPR spectra, subtraction procedures, linear regression analysis and characterization of normal data distribution were performed using Microcal Origin software and GraphPad Prism4. Parametric two-tailed or one-tailed t-test, as indicated, was applied using GraphPad software, after verification of normal distribution of values. Data are expressed as the mean +/− standard error (SE). P values <0.05 was accepted as significant.

## Results

### Clinical Characteristics and Endothelial Function of the Study Population

The clinical characteristics of all study subjects (n = 50) are shown in Table 1. Mean age was 24.7+/−0.8 years, the male/female ratio was 46/54% and all had normal BMI (22.7+/−0.5); all were free of diabetes or hypertension; the cardiovascular risk profile according to the SCORE level [19] was below 1% for all; levels of glycated Hb, homocysteine, and lipid profile are shown in Table 1.

**Table 1:** Clinical and biological characteristics of the study subjects.

Clinical and biological characteristics	Number and Mean values ± SE	Normal values
Age, (y)	24.7±0.8	
Male, n (%)	23 (46%)	
Female, n (%)	27 (54%)	
Body mass index, BMI (kg/m^2^)	22.7±0.5	18.5–24.9
Weight (kg)	67.7±1.7	
Hypertension, n (%)	0	
Diabetes, n (%)	0	
Smoking, n(%)	3 (6%)	
Sport, n (%)	28 (56%)	
Resting heart rate (beats/min)	66±1	60–80
Systolic blood pressure, (mmHg)	119.4±1.7	<140
Diastolic blood pressure, (mmHg)	74.6±1.4	<90
Hb, (g/dl)	13.79±1.57	11–18
Hb A1c, (%)	5.2±0.04	<6.0
Fasting glucose, (mg/dL)	94.1±1.5	<100
Hematocrit, (%)	40.5±1.0	34–54
Creatinine, (mg/dl)	0.82±0.03	0.2–1.0
Urea, (mg/dl)	28±1	<50
Homocysteine, (µmol/L)	11.98±0.9	<15
C-Reactive Protein, (mg/dl)	0.39±0.12	<1
Total cholesterol, (mg/dl)	168.6±3.9	<200
HDL cholesterol, (mg/dl)	60.7±2.5	>40
LDL cholesterol, (mg/dl)	87.8±3.7	<115
Total/HDL cholesterol	3.08±0.78	<5
Triglycerides, (mg/dl)	92.8±5.5	35–150

The mean value of FRHI, the reactive hyperemia index calculated as described in Materials and Methods, was 0.58±0.06 (range −0.22 to 1.55) for the whole group; when the analysis was done separately for males and females, the mean value of FRHI was 0.40±0.08 in males (n = 23 ) and 0.73±0.08 in females (n = 27; P<0.05).

### Measurements of RBC HbNO Level by EPR Spectroscopy Using Subtraction of Free Radical Signal

**Figure 3:**
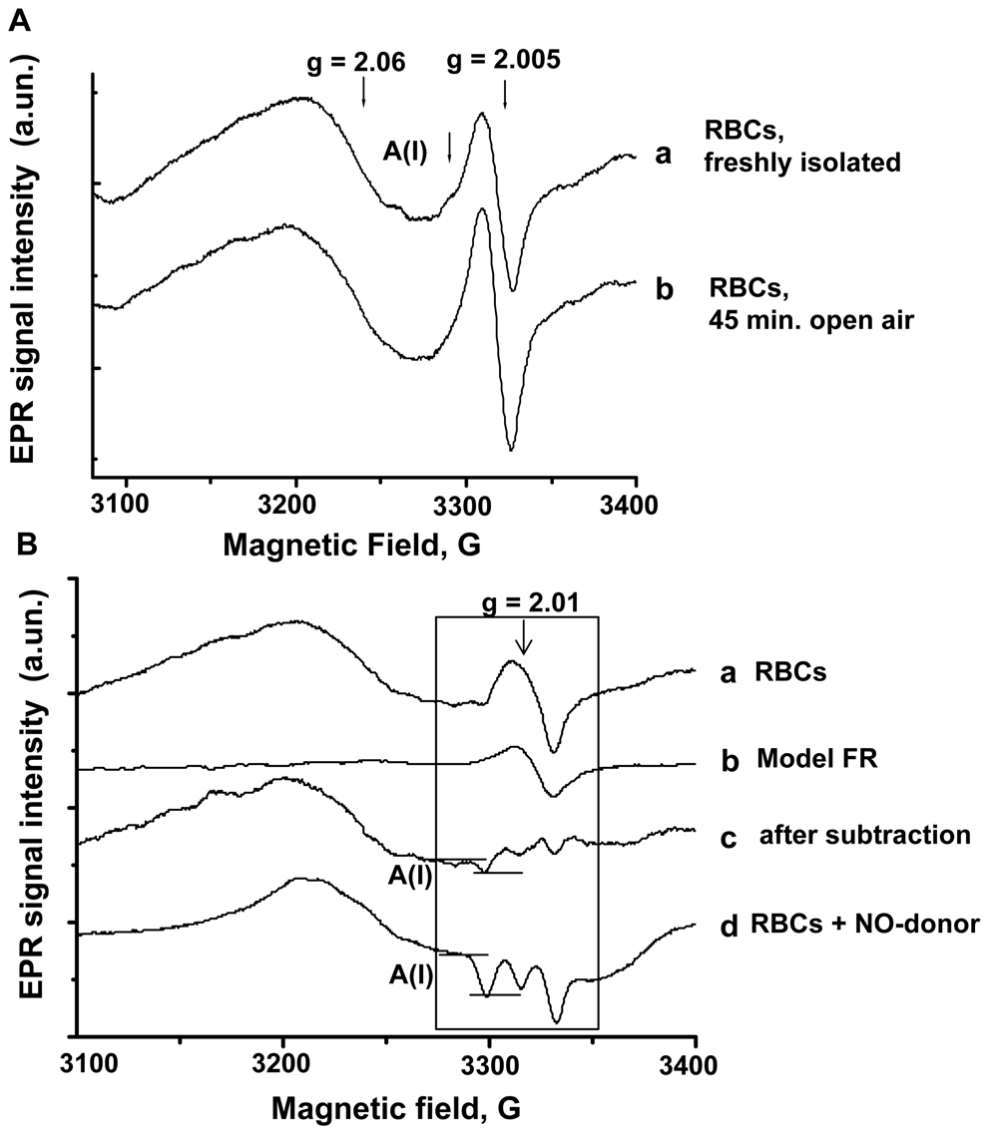
Quantification of Hb-NO from human venous erythrocytes. (A) Typical EPR spectra of RBCs, frozen after blood centrifugation (a), and after 45 minutes of exposure to air (b) (diapason: 3100–3400 Gauss). (B) Final EPR spectrum of RBCs (c, gain = 6.2×10^4^), after subtraction of the model spectrum of free radicals (b), divided by the correspondent proportional scale factor, from the EPR spectrum of the initial sample frozen immediately (a). Typical EPR spectra of RBCs, frozen after incubation under low O_2_ level with the NO-donor, Dea-NONOate (50 µmol/L; c, gain = 3.1×10^4^). A peak-to- peak amplitude of hf component, A(I), of the HbNO triplet structure attributed to 5-coordinate α-HbNO was used for quantitation.

The EPR spectra from human erythrocytes are typically composed of superposed EPR signals from different paramagnetic centres in the region 3000–3400 Gauss (g ∼ 2.06–1.9), where the HbNO EPR signal is observed. Figure 3A illustrates typical EPR spectra before subtraction from the sample of RBCs freshly isolated at venous oxygen level and frozen immediately (a), or stored in open air for 45 minutes (b). The most prominent signals are a residual copper(II)-protein signal (g value ∼ 2.06) and the sharp signal of protein-centred free radical (FR) species (g ∼ 2.005) with individually fluctuating peak-to-peak width ∼ 16–18 G [17], [18]. The latter, in particular, overlapped with the hf structure of HbNO signal (A(I)), as indicated in Figure 3A. Accordingly, to reveal and quantitate the HbNO level in human RBCs, we used a subtraction procedure as described in Materials and Methods after determination of the spectrum component corresponding to erythrocytic protein-centred FR from each subject. The results of subtraction are shown in Figure 3B (spectrum c), clearly revealing the first component of the hf structure (A(I)). This exactly aligns with the A(I) component obtained from RBCs treated ex vivo with NO donors as described in Materials and Methods, where the amplitude (between the two horizontal bars) was increased in proportion with the concentration of exogenous NO applied (see Figure S1 for a full dose-response). The amplitude of A(I) of the HbNO hf structure was used for quantification as proposed previously in animal models [18], [20]. The actual HbNO concentration in RBCs was determined by comparison with a calibration curve (Figures **S**1A and S1B).

With this individualized subtraction method, the mean resulting concentration of corpuscular HbNO in the study group was measured at 219+/−12 nmol/L (range 86–473 nmol/L, N = 50). When compared between genders, mean HbNO was 201+/−16 nmol/L in males (n = 23) and 235+/−18 nmol/L in females (n = 27) (P>0.05); however, we observed a significant difference among female volunteers taking (or not) contraceptive pills, i.e. 199+/−19 nmol/L (n = 15) and 279+/−29 nmol/L (n = 12), respectively (P<0.05); when only volunteers without contraception were considered, females had significantly higher HbNO than males (P<0.05). Using univariate model analysis, we did not find any significant correlation between erythrocyte HbNO concentrations and mean corpuscular hemoglobin concentrations, or hematocrit in the whole cohort (Figures S3A and S3B).

### Linear Regression Analysis between HbNO Level in RBCs from Venous Blood and FRHI

**Figure 4:**
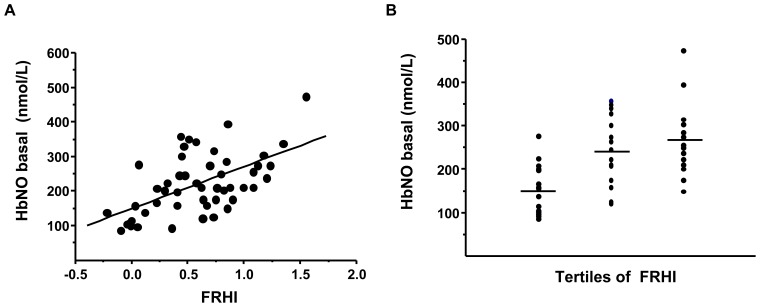
Hb-NO levels correlate with endothelial function in normal subjects. (A) Linear regression analysis between basal HbNO level in RBCs isolated from venous blood and FRHI calculated as described in Methods (r = 0.58, P<0.0001). (B) Analysis of HbNO levels in RBCs isolated from venous blood of subjects distributed in different tertiles of FRHI as measured in the same arm; one-way analysis of variance (ANOVA) shows significant difference of mean values between groups (P = 0.0002).

**Figure 5:**
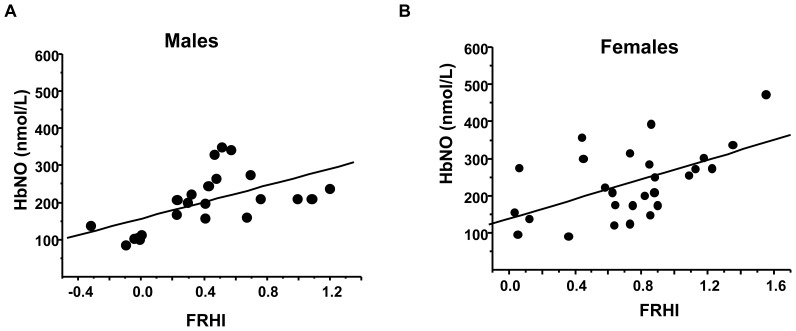
Hb-NO levels correlate with reactive hyperemia both in males and females. Linear regression analysis between basal HbNO level (nmol/L) in RBCs isolated from venous blood of male (A) and female (B) subjects and FRHI calculated as described in Methods (r = 0.57; P<0.005; n = 23 and r = 0.55; P<0.005; n = 27 respectively).

The corpuscular concentrations of HbNO were significantly correlated with FRHI in the entire cohort (r = 0.58, P<0.0001, see Figure 4A). Similar significant correlations were found between HbNO and the other reactive hyperemia indexes (RHI and ln_RHI) calculated as detailed in Methods (results not shown). Separation of FRHI data by tertiles coincided with correspondingly increased HbNO levels (Figure 4B). HbNO also significantly correlated with FRHI in male and female volunteers when analyzed separately (Figure 5A and B).

**Figure 6:**
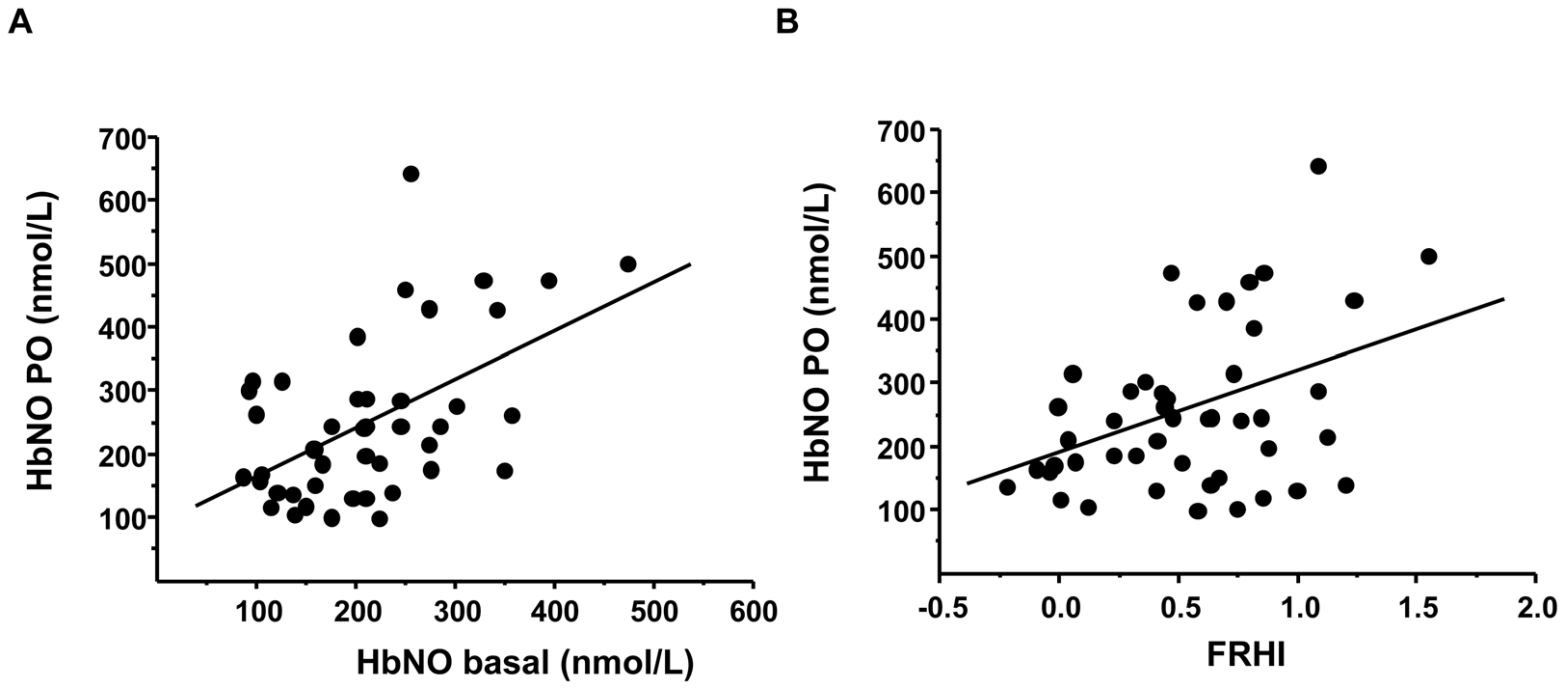
Post-occlusive Hb-NO correlates with resting Hb-NO and reactive hyperemia. Linear regression analysis between HbNO level in RBCs isolated from brachial vein during the post-occlusion (PO) period (maximum HbNO value at 1 or 2 minutes PO) and basal HbNO level (A; r = 0.53; n = 46; P = 0.0001) or FRHI (calculated as described in Methods) (B; r = 0.4; n = 46; P = 0.004). The line represents the least-square regression line.

We also observed dynamic changes of HbNO levels measured in RBCs isolated 1 and 2 minutes after cuff deflation (i.e. during reactive hyperemia). The maximal post-deflation HbNO concentration was significantly increased to 120+/−8% of basal levels (P<0.05; N = 46). This maximal post-deflation HbNO is also significantly correlated with basal HbNO level and FRHI measured simultaneously in the same subject (Figure 6A and 6B).

## Discussion

Given the pleiotropic signalling functions of nitric oxide in physiology and pathology, quantitative measurements of circulating bioactive NO in humans is highly desirable. HbNO can accumulate in erythrocytes from a number of sources including from eNOS activity in the vasculature and subsequent release of NO from erythrocytes was even proposed to mediate vasodilatory effects in hypoxic tissues through various mechanisms (reviewed in [21]). In this study, we developed an improved method of detection of HbNO and examined the correlation between erythrocytic HbNO level and endothelial function in human subjects.

The EPR study of blood HbNO level was successfully used in animal models over the last decade and provided unique information about systemic NO bioavailability in correlation with eNOS activity in isolated vessels [18], [20]. However, its applicability to humans has been hampered by differences in intensity of the EPR signals overlapping with the HbNO EPR spectra. This was mostly due to larger intensity of the EPR signals of Cu-containing proteins (ceruloplasmin) in plasma and protein FRs that prevented accurate analysis of HbNO EPR signals in human blood. Our approach is based on rapid RBCs isolation at proper O_2_ pressure from venous blood, followed by subtraction analysis of the EPR spectra using the spectrum component corresponding to protein FRs, as determined individually. This allowed “unmasking” of the triplet hf structure of 5-coordinate α-HbNO and its quantification in RBCs. We then applied this method to assess basal and reactive hyperemia-induced changes in HbNO in human RBCs.

In our cohort of normal volunteers (N = 50), basal venous erythrocyte HbNO levels ranged from 86–473 nmol/L, with a normal distribution and mean value of 219+/−12 nmol/L This is close to, albeit slightly higher than HbNO levels previously measured by EPR in whole human blood, where values were reported not to exceed 200 nmol/L [17]. Another study of HbNO using ozon-based chemiluminescent detection of NO gas released from the reaction of tri-iodide with hemoglobin freshly isolated from human venous RBCs in 5 normal volunteers gave values of 0.002+/−0.0005% of haemoglobin tetramer concentration [22]. This corresponds to an estimated HbNO concentration of about 100 nmol/L in RBCs based on a 5 mmol/L hemoglobin concentration in red cells. Again, our estimation is higher, for several possible reasons; (i) in contrast to the latter study, we used RBC that were frozen immediately after centrifugation, without any washing step or further processing; this may have better preserved nitrosyl-hemoglobin adducts, in particular the 5-coordinate α-HbNO which has low stability, whereas these may have been lost in the processing of samples for chemiluminescence analysis; (ii) our study involved a larger group of subjects (N = 50); (iii) compared with previous EPR studies, we analysed the erythrocytes fraction directly, as opposed to whole blood, and improved the sensitivity of our analysis by subtracting the FR component determined individually; this is probably critical, given the expected inter-individual variability in protein FR content.

Indeed, we observed differential reactivity of anti-oxidants with protein-centred FRs among our normal volunteers. Formation of the radicals on peptides and proteins in processes of the protein oxidation has been intensively investigated recently [23]. In particular, EPR signals of globin-based radicals with g ∼ 2.005 were detected in human blood under oxidative conditions [24]. At least two different forms of protein-based free radicals were observed in human arterial and venous blood of the same individuals [17]. We have found that the form and line width of the signal slightly varied from subject to subject. This justified our approach to determine the free radical signal component on an individual basis, using a subtraction method of compared spectra with/without an antioxidant. Our observation of anti-oxidant or pro-oxidant effects of ascorbic acid on erythrocyte FRs among different subjects was unexpected, given the conventional antioxidant properties of vitamin C under normal biological conditions [25]. In fact, pro-oxidant effects of vitamin C were demonstrated previously *in vivo* after i.p. or i.v. administration resulting in high plasma concentrations; this resulted in the formation of ascorbate radicals and hydrogen peroxide in extracellular fluids resulting in oxidant stress [26], [27]. Although the exact biochemical reaction(s) has(−ve) not been resolved, cupric or ferric ions of metal-centered proteins (ceruloplasmin, for example) could easily be reduced by ascorbate with ensuing ROS formation by reduced transition metal ions. This may well be reproduced with millimolar concentrations of ascorbic acid as we added to pelleted erythrocytes in our protocol. Steady-state levels of formed radicals will then be determined by the individual redox capacity and amount of antioxidant or “detoxifying” enzymatic systems in the erythrocytes membranes, including superoxide dismutase (SOD), glutathion peroxidase and catalase [28], [29], [30].

Lower “detoxifying” capacity would then result in excessive ROS production after ascorbic acid, accounting for the pro-oxidant effect. Importantly, we verified that subtraction of the model spectra derived from anti- vs. pro-oxidant effects of ascorbic acid did not introduce a systematic bias in the measurement of the HbNO signal, e.g. in both cases mean line width of the model spectra was not significantly different, avoiding systematic errors on the measured amplitude of the first hf component.

The HbNO levels observed by us *ex vivo* also did not significantly correlate with hematocrit or mean corpuscular Hb concentration, as could have been anticipated from a previous study of nitric oxide diffusion into isolated RBCs *in vitro* [31]. The very small concentration of NO compared to corpuscular Hb probably made other factors, particularly interaction with RBC free radicals, more limiting.

With our original subtraction method, we were able to measure quantitative changes in HbNO during post-occlusion hyperemia; indeed, we observed dynamic increases of the erythrocytic HbNO signal to (120+/−9%) at 1–2 minutes post-occlusion, corresponding to the maximal hyperemia measured by tonometry. Of note, both post-occlusion and basal HbNO values were significantly correlated with FRHI, and post-occlusion HbNO also varied linearly with basal HbNO in the same subjects. This suggests that basal HbNO may reflect vascular function. Our observation of a significant, albeit moderate correlation (r^2^ = 0.34) is perhaps not unexpected, given the previous observation that despite the well-established role of endothelial nitric oxide as mediator of shear stress-induced vasodilation [32], only 46% of the RH signal was inhibited by administration of the nitric oxide synthase inhibitor, L-NAME into the brachial artery prior to tonometric measurement [10]. Accordingly, the moderate correlation probably reflects the involvement of other, NO-independent vasodilating factors. Also, NO may be differentially distributed between the abluminal and luminal space, where a number of variables may additionally affect its fate until it reaches the erythrocyte and reacts with Hb. These may include the diffusion distance between the endothelial membrane and circulating erythrocytes at the vessel centre, as well as barriers at the erythrocyte membrane itself [33]. Finally, the HbNO signal in RBC may partially result from NO production in the erythrocyte itself, either from local eNOS activity, as was recently proposed [34], or nitrite reductase activity of Hb [35]. While the potential involvement of these different sources is currently being investigated, our present results suggest that, regardless of the source, erythrocyte HbNO correlates with vascular function. Even if HbNO integrates the cumulated influences of prevailing oxidant stress and other factors affecting eNOS expression/activity from other sources, these are likely to similarly affect endothelial NO-dependent signalling; whether HbNO may then be a relevant biomarker reflecting the integrity of endothelial NO-dependent function will need to be assessed in future cohorts of normal and diseased subjects with cardiovascular risk factors, as was done for FRHI in recent epidemiological studies [8], [9]. In the present cohort of normal volunteers, at least, we found that basal venous HbNO was higher in young females (free of contraceptive pills) than males, which correlates with higher RH in the former; this would be in line with the well-known lower cardiovascular risk in pre-menopausal women. The fact that HbNO were lower in young females taking contraceptive pills, independently of differences in BP or lipid profile, may indicate a toxicity of hormonal treatment on NO bioavailability and/or endothelial function. This deserves further studies in larger cohorts.

In conclusion, our study demonstrates quantitative measurements of 5-coordinate α-HbNO in human venous erythrocytes, its dynamic physiologic regulation and correlation with endothelial function assessed by tonometric measurement of reactive hyperemia in a cohort of normal volunteers. This opens the way to better understanding of in vivo determinants of NO bioavailability and its regulation in pathophysiologic states, as will be determined in future studies.

## Supporting Information

Figure S1 **Hb-NO dose-dependently increases in erythrocytes after exposure to exogenous NO donor.** (A) EPR signals of HbNO formed in intact RBCs treated with increasing concentrations of nitrite in presence of Na_2_S_2_O_4_ (sodium dithionite, 20 mM) in anaerobic condition. (B) Calibration curve for the quantification of HbNO concentrations, as obtained from the spectra illustrated in (A). Parameters of linear regression were r = 0.99; P = 0.001; N = 4.
(PDF)

Figure S2 **Reverse subtraction procedure in erythrocytes exhibiting increased free radicals after ascorbic acid.** Typical EPR spectra of free radicals in RBCs from a subject in which Ascorbic Acid increased free radicals. Shown are the spectra of RBCs left for 45 min in open air at room temperature and treated with solvent (a), or with ascorbic acid (AA) for the last 15 minutes (b). The model spectrum of free radicals was obtained after inverse subtraction (b–a) of the EPR spectrum of RBC treated with vehicle (a) from the spectrum of RBCs treated with ascorbic acid (b).
(PDF)

Figure S3 **Absence of correlation of Hb-NO with blood hemoglobin concentration or hematocrit.** Linear regression analysis between basal HbNO level (nmol/L) in RBCs isolated from venous blood and (A) mean concentration of corpuscular Hb (r = 0.1, P = 0.6, n = 49); or (B) hematocrit (r = 0.1, P = 0.3, n = 49).
(PDF)
