# Supplementary figures and images for: Nitrosylated Hemoglobin Levels in Human Venous Erythrocytes Correlate with Vascular Endothelial Function Measured by Digital Reactive Hyperemia
Source: PLoS One. 2013 Oct 10;8(10):e76457. doi: 10.1371/journal.pone.0076457 (PMC3794924; doi:10.1371/journal.pone.0076457)

Figure S1

A

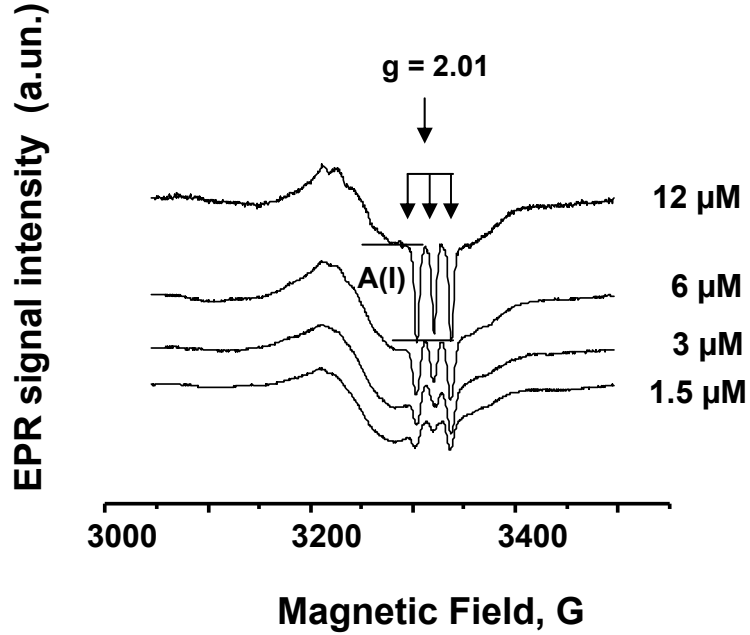

B

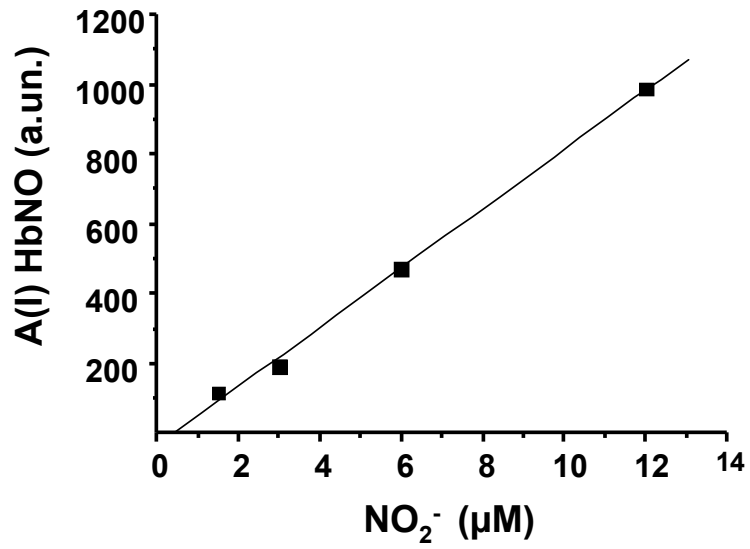

Supplement: Figure S1 — Hb-NO dose-dependently increases in erythrocytes after exposure to exogenous NO donor. (A) EPR signals of HbNO formed in intact RBCs treated with increasing concentrations of nitrite in presence of Na2S2O4 (sodium dithionite, 20 mM) in anaerobic condition. (B) Calibration curve for the quantification of HbNO concentrations, as obtained from the spectra illustrated in (A). Parameters of linear regression were r = 0.99; P = 0.001; N = 4. (PDF) [file pone.0076457.s001.pdf]

Figure S2

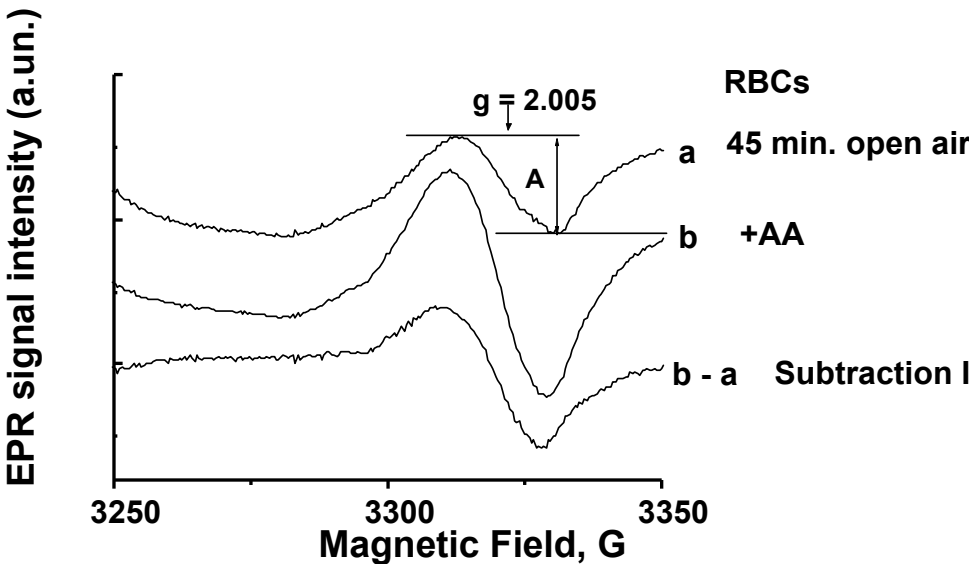

Supplement: Figure S2 — Reverse subtraction procedure in erythrocytes exhibiting increased free radicals after ascorbic acid. Typical EPR spectra of free radicals in RBCs from a subject in which Ascorbic Acid increased free radicals. Shown are the spectra of RBCs left for 45 min in open air at room temperature and treated with solvent (a), or with ascorbic acid (AA) for the last 15 minutes (b). The model spectrum of free radicals was obtained after inverse subtraction (b–a) of the EPR spectrum of RBC treated with vehicle (a) from the spectrum of RBCs treated with ascorbic acid (b). (PDF) [file pone.0076457.s002.pdf]

Figure S3

A

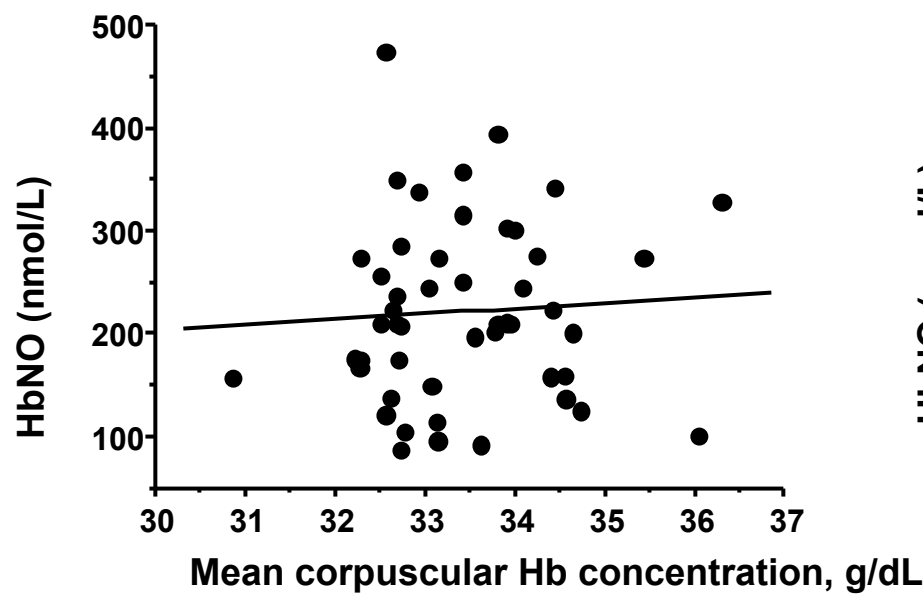

B

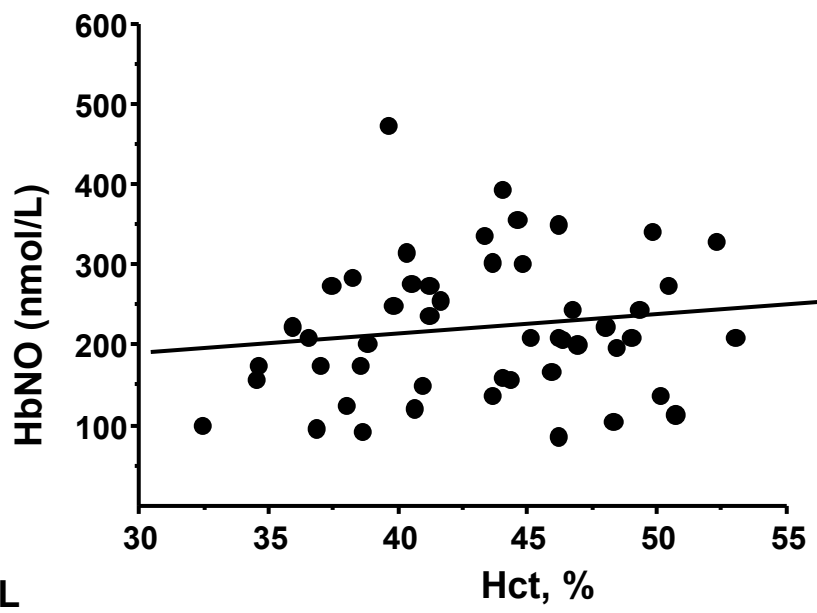

Supplement: Figure S3 — Absence of correlation of Hb-NO with blood hemoglobin concentration or hematocrit. Linear regression analysis between basal HbNO level (nmol/L) in RBCs isolated from venous blood and (A) mean concentration of corpuscular Hb (r = 0.1, P = 0.6, n = 49); or (B) hematocrit (r = 0.1, P = 0.3, n = 49). (PDF) [file pone.0076457.s003.pdf]
